# Evaluation and identification of advanced lentil interspecific derivatives resulted in the development of early maturing, high yielding, and disease-resistant cultivars under Indian agro-ecological conditions

**DOI:** 10.3389/fpls.2022.936572

**Published:** 2022-09-08

**Authors:** Mohar Singh, Sanjeev Kumar, Reena Mehra, Salej Sood, Nikhil Malhotra, Reena Sinha, Sonika Jamwal, Vikas Gupta

**Affiliations:** ^1^ICAR-National Bureau of Plant Genetic Resources, Shimla, India; ^2^Department of Genetics and Plant Breeding, Sher-e-Kashmir University of Agricultural Sciences and Technology, Jammu, India; ^3^International Center for Agricultural Research in Dry Areas-Food Legume Research Platform, Bhopal, India; ^4^ICAR-Central Potato Research Institute, Shimla, India; ^5^Advanced Centre for Rainfed Agriculture Dhiansar, Sher-e-Kashmir University of Agricultural Sciences and Technology, Jammu, India

**Keywords:** lentil, interspecific derivatives, agronomic performance, cultivars, biotic stresses, agronomy

## Abstract

The genetic base revealed by pedigree records of the majority of released cultivars appears to be narrow in major pulse crops, including lentils, because of the frequent use of the same parents and their derivatives in crop improvement programs. Therefore, corrective measures are needed to widen the genetic base by involving the genetic resources of a distinct gene pool. In this direction, rigorous efforts were made to introgress wild *Lens* taxa, *L. culinaris* ssp. *orientalis*, and *L. ervoides* into the backgrounds of cultivated varieties. Subsequently, genetic materials were advanced through the single seed descent method of breeding along with a rapid generation advancement (normal and off-season) approach. Two F_10:11_ interspecific derivatives of lentils were evaluated in augmented block design at two locations, *viz.* International Centre for Agricultural Research in Dry Areas (ICARDA) and Sher-e-Kashmir University of Agricultural Sciences and Technology (SKUAST), India. The analysis of variance showed remarkable variability for all target characters at both locations. The heritability estimates were high, and correlation analysis exhibited a significant association between the majority of traits assessed at ICARDA and SKUAST, India. Further, SKUAST identified the most promising lines as “Jammu Lentil 144” and “Jammu Lentil 71.” These derivatives were further validated separately for their agronomic potential and resistance against major biotic stresses. The results revealed that Jammu Lentil 144 and Jammu Lentil 71 produced 16.65 and 9.40% more seed yield than local and national checks, including earliness, by 25 and 15 days, respectively. These promising interspecific derivatives were also found to be resistant to fusarium wilt, root rot, pod borer, and aphid infestations. The standard agronomy of these cultivars has also been assessed consecutively for 2 years at SKUAST. Overall, the pre-breeding efforts have resulted in the development of early maturing, high-yielding, and disease-resistant lentil cultivars for the Jammu region of India.

## Introduction

The cultivated lentil (*Lens culinaris* ssp. *culinaris*) Medikus is a true diploid (2*n* = 2*x* = 14) annual self-pollinating grain legume crop. It encompasses two major groups selected based on distinct morphological traits, the small-seeded (*microsperma*) and large-seeded (*macrosperma*), along with *L. culinaris* ssp. *orientalis*, presumed wild progenitor taxa (Zohary, 1972). The other species are *L. culinaris* ssp. *odemensis*, *L. nigricans*, *L. tomentosus*, and *L. ervoides*. Lentil is an important crop in the Indian sub-continent, the Middle East, Southern Europe, and Eastern and Northern Africa. The crop has several useful traits of interest: it is highly valued for its good protein quality (22–35%), minerals, and vitamins for human nutrition (Bhatty, 1988). Globally, the crop ranks sixth in terms of production among major pulses (FAO, 2019). In the past 2–3 decades, lentil genetic improvement activities have relied on local traditional landraces. Of late, the high-yielding cultivars were developed from a few numbers of improved landraces through pure line selection following hybridization between genotypes adapted to niche environmental conditions. These improved cultivars have some superiority over local ecotypes in terms of their yield potential and adaptation against major stresses like fusarium wilt, powdery mildew, root rot, cold, drought, and others (Gupta and Sharma, 2006; Singh et al., 2014; Malhotra et al., 2019). Like other pulse crops, the lentil has not earned many genetic gains in yield due to a lack of combinations of potential genes and alleles for higher productivity, including major biotic and abiotic stresses, which are scattered among various sources, including wild (Rubio Teso et al., 2022). So, exploitation of the unadapted gene pool for broadening the genetic base of cultivated varieties and selection of promising derivatives among enhanced progenies would be fruitful criteria for developing elite cultivars. In fact, only a few reports are available to undertake systematic studies on lentil interspecific hybridization and subsequent generation advancement up to the level of improved cultivars. Therefore, pre-breeding and over-location evaluation of advanced interspecific derivatives is needed to assess their performance and identify the basis of genotype adaptation. This is imperative because the performance of derivatives can vary from one environment to another, and genotypes that are best in one environment may not be superior in another environment due to genotype × environment (GE) interactions (Makumbi et al., 2015). Hence, the objectives of this study were designed to assess the comparative performance of advanced lentil interspecific derivatives under two agroecological conditions in India and to identify the most promising derivatives for developing early-maturing, high-yielding, and disease-resistant lentil cultivars.

## Materials and methods

### Plant material

**FIGURE 1:**
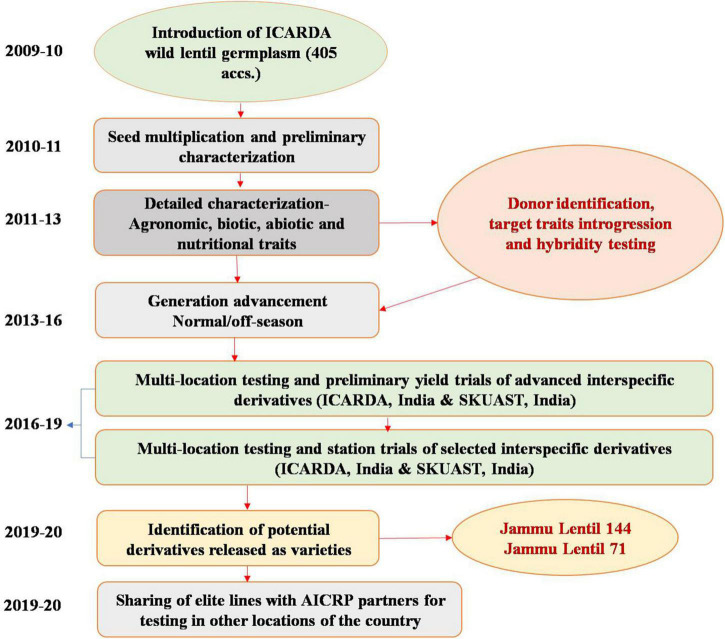
A holistic approach for broadening the genetic base of the domesticated gene pool for developing improved cultivars in lentils.

The plant material comprised two F_10:11_ interspecific populations of lentil, which were originally derived from ILL8006 (*L. culinaris* ssp. *culinaris*) × ILWL62 (*L. culinaris* ssp. *orientalis*) and ILL10829 (*L. culinaris* ssp. *culinaris*) × ILWL30 (*L. ervoides*) during the winter seasons of 2011–2012 and summer 2012 for transferring target traits (earliness, pod number, and resistance against prevailing biotic stresses). The true-to-type hybridity of these interspecific derivatives was confirmed through morphological and molecular (ISSR) markers (Singh et al., 2013). During the generation advancement period (F_2_ onward), transgressive segregations and fruitful heterosis of these interspecific derivatives were also estimated as desirable traits of interest (Singh et al., 2017). A total of 137 interspecific derivatives of cross ILL8006 × ILWL62 and 176 of ILL10829 × ILWL30 were advanced through the single seed descent (SSD) breeding method (Johnson and Bernard, 1962) to attain a high level of homozygosity for accelerating the breeding process. The holistic approach followed to undertake global wild lentil species introduction from International Centre for Agricultural Research in Dry Areas (ICARDA), their seed multiplication, characterization, evaluation, and introgression for broadening the genetic base of domesticated gene pool developing improved cultivars is presented in Figure 1.

### Agronomic evaluation and experimental design

The advanced generations (F_10:11_) of ILL10829 × ILWL30 and ILL8006 × ILWL62 were assessed for agronomic performance in varied agroecological regions of India, *viz.* International Centre for Agricultural Research in Dry Areas (ICARDA) Pulse Research Platform at Amlaha, Bhopal (23.06°N, 77.05°E, 499 amsl) and Sher-e-Kashmir University of Agricultural Sciences and Technology (SKUAST), Jammu (32.68°N, 74.82°E, 281 amsl) in India during winter seasons (2017–2018 and 2018–2019 at ICARDA; and 2016–2017, 2017–2018, and 2018–2019 at SKUAST). These interspecific derivatives were grown in field trials conducted at two agroecological locations (Table 1). The experiments were conducted in Augmented Complete Block Design (ACBD). Each entry was planted in three rows of 3 m in length, 30 cm apart, and spaced at 10 cm. In all the experiments of each location, three respective regional/local checks were also used to compare the performance of interspecific derivatives. Furthermore, SKUAST also conducted another experiment on selected interspecific derivatives from which potential derivative lines 144 and 71 were selected and named “Jammu Lentil 144” and “Jammu Lentil 71.” The trials on these selected lines were conducted using a Complete Randomized Block design (CRBD) replicated thrice. The observations were recorded on important agronomical traits such as days to 50% flowering (DF), days to 80% maturity (DM), the number of pods plant^–1^ (NPPP), 100-seed weight (SW), and seed yield plant^–1^ (SYPP) as per minimal descriptors developed by ICAR-National Bureau of Plant Genetic Resources (Mahajan et al., 2000). One pre-sowing irrigation was applied to ensure proper seed germination, and no other irrigation was given during the whole cropping period. Good weed management operations were carried out using mechanical weeding and hoeing to keep the experimental area free from any unwanted weeds.

**TABLE 1:** Agro-climatic description of the locations where advanced lentil interspecific derivatives were evaluated during the study period.

Location	Latitude	Longitude	Elevation (m)	Mean rainfall (mm)	Temperature (°C)		Soil type
						
					Min	Max	
ICARDA, India	23.06°N	77.05°E	499	1,207	3.49	40.30	Black cotton
SKUAST, India	32.68°N	74.82°E	281	1,300	8.30	32.20	Sandy loam

### Screening against major biotic stresses

#### Fusarium wilt and root rot

Disease incidence percentage (fusarium wilt and root rot) was recorded during the winter seasons of 2016–2017, 2017–2018, and 2018–2019. Fusarium wilt of lentils is caused by *Fusarium oxysporum*. f. sp. *lentis* and root rot by *Sclerotium rolfsii*. The incidence percentage was calculated using the below formula:


$$\mathrm{Percent}\,\mathrm{disease}\,\mathrm{incidence}=\frac{\mathrm{Number}\,\mathrm{of}\,\mathrm{plants}\,\mathrm{showing}\,\mathrm{wilting}\,\mathrm{symptoms}}{\mathrm{Total}\,\mathrm{number}\,\mathrm{of}\,\mathrm{plants}}\times 100$$

Based on the aforementioned formula, wilt incidence has been calculated as described by Biswas and Jubayer Ali (2017). The wilt incidence was recorded at 30-day intervals till harvest. In each plot, three rows, each 3 m long, were chosen arbitrarily. Plants in each row were examined, and the number of plants showing yellowing or wilting vascular symptoms was noted. Disease incidence was expressed as the percentage of affected plants, counted in three rows by the total number of plants. The percent disease incidence in each treatment was calculated using the following formula:


$$\mathrm{Percent}\,\mathrm{wilt}\,\mathrm{incidence}=\frac{\mathrm{Number}\,\mathrm{of}\,\mathrm{plants}\,\mathrm{wilted}}{\mathrm{Total}\,\mathrm{number}\,\mathrm{of}\,\mathrm{plants}}\times 100$$

#### Lentil pod borer [*Etiella zinckenella* (Treitschke)]

Ten plants were selected randomly, where the number of damaged pods and the total number of pods were counted at harvest during three consecutive years (2016–2017, 2017–2018, and 2018–2019). Pods with the faucal material of *E. zinckenella*, webbing, and exit holes of the lentil pod borer were considered damaged pods (Kumar and Yadav, 2018). Percent pod damage was calculated using the formula:


$$\mathrm{Percent}\,\mathrm{pod}\,\mathrm{damage}=\frac{\mathrm{Number}\,\mathrm{of}\,\mathrm{damaged}\,\mathrm{pods}}{\mathrm{Total}\,\mathrm{number}\,\mathrm{of}\,\mathrm{pods}}\times 100$$

#### Lentil aphid [*Aphis craccivora* (Koch.)]

Both nymphs and adult aphids damage the crop by sucking sap from tender leaves and shoots, thus retarding plant growth. Severe attacks result in stunted growth, reduced seed size, and stunted pods. The lentil aphid appeared 75–85 days after sowing (DAS). Data were recorded at 15 days intervals in ten plants selected randomly from each entry covering the whole plot. The assessment of infestation by aphids was done using the 0–9 rating scale of Kumari et al. (2009) during 2016–2017, 2017–2018, and 2018–2019.

### Statistical analysis

The analysis of variance (ANOVA) of Augmented Complete Block Design was carried out using the R package “augmented RCBD” (Aravind et al., 2020). The linear mixed models were implemented in lmer from package lme4 of R using REML to calculate BLUEs and BLUPs and estimate the variance components (R Core Team, 2020). The adjusted means of all the quantitative traits, *viz.* days to 50% flowering, days to 80% maturity, number of pods plant^–1^, seed weight, and seed yield plant^–1,^ were used to estimate principal component analysis (PCA), cluster analysis, and correlations. The PCA and correlation were studied to assess the overall contribution of studied traits to significant variations in lentil interspecific derivatives using gplot and cor function in R, respectively. The R package “corrplot” was used to depict correlation plots. The standard errors for the critical comparisons were analyzed using box plots. The numerical data were also subjected to basic statistical analysis using MS Office Excel Software.

## Results

### Evaluation of lentil interspecific derivatives

The analysis of variance revealed significant variation among blocks and treatments for days to 50% flowering, days to 80% maturity, the number of pods plant^–1^, 100-seed weight, and seed yield plant^–1^ in the cross ILL10829 × ILWL30 at ICARDA (Table 2). Likewise, the treatments showed significant differences for the majority of characters for the cross of ILL8006 × ILWL62 except for days to 50% flowering and the number of pods plant^–1^ Similarly, in SKUAST, the analysis of variance revealed significant differences among treatments for all the traits in both the crosses except for days to 50% flowering and days to 80% maturity in the cross ILL8006 × ILWL62. Further, the descriptive statistical parameters indicated a wide range of variation with respect to important agronomical characters (Table 3). However, the distribution of these traits was highly skewed and significant for all the characters in both the crosses at ICARDA except the 100-seed weight of cross ILL8006 × ILWL62. Likewise, the breeding lines in both the interspecific crosses revealed significant variation with the normal distribution of all the traits at SKUAST (Table 3).

**TABLE 2:** Analysis of variance of lentil interspecific derivatives for seed yield and its important component traits at ICARDA and SKUAST, India.

ICARDA										

Cross ILL10829 × ILWL30						Cross ILL8006 × ILWL62				
	
Source	DF	DM	NPPP	SW	SYPP	DF	DM	NPPP	SW	SYPP
Block (ignoring Treatments)	110.33**	72.64**	4,992.69**	0.12**	2.85**	109.40*	50.62 ns	4,182.48 ns	0.29*	3.54 ns
Treatment (eliminating Blocks)	87.28**	101.48**	4,183.68**	0.12**	2.42**	54.26 ns	74.84*	6,701.51 ns	0.41**	3.16*
Treatment (Check)	3,351.61**	3,395.11**	3,940.46**	0.99**	7.99**	608.25**	1,317.56**	8,088.33 ns	0.15 ns	6.56*
Treatment (Test and Test vs. Check)	20.66 ns	34.26*	4,188.65**	0.10**	2.31**	31.17 ns	23.06 ns	6,643.72 ns	0.42**	3.01*

**SKUAST**										

**Cross ILL10829 × ILWL30**						**Cross ILL8006 × ILWL62**				
	
**Source**	**DF**	**DM**	**NPPP**	**SW**	**SYPP**	**DF**	**DM**	**NPPP**	**SW**	**SYPP**

Block (ignoring Treatments)	133.94**	113.97**	16,687.14**	12.51**	81.43**	271.60 ns	84.89 ns	57,615.96**	0.73**	108.48**
Treatment (eliminating Blocks)	94.10**	79.86**	5,389.83**	0.44**	8.57**	108.72 ns	69.53 ns	6,997.29**	0.17**	10.72**
Treatment (Check)	2,754.67**	3,138.00**	6,453.36**	0.32**	11.43**	1,483.07**	1,576.05**	6,453.65**	0.30**	9.75**
Treatment (Test and Test vs. Check)	40.53**	18.28**	5,368.42**	0.44**	8.51**	61.33 ns	17.58 ns	7,016.03**	0.17**	10.75**

*Significant at p = 0.01, **Significant at p = 0.05, ns, non-significant.

**TABLE 3:** Summary of descriptive statistical parameters for important agro-morphological traits at ICARDA and SKUAST, India.

ICARDA														

Cross ILL10829 × ILWL30								Cross ILL8006 × ILWL62						
	
Trait	Min	Max	Mean	SE	SD	Skewness	Kurtosis	Min	Max	Mean	SE	SD	Skewness	Kurtosis
DF	47.00	92.37	54.96	0.39	4.82	4.87**	31.70**	52.00	79.13	68.86	0.63	5.45	−0.64*	3.77 ns
DM	89.03	138.62	96.18	0.45	5.52	3.33**	25.01**	90.31	134.00	100.61	0.79	6.89	1.80**	8.73**
SYPP	1.21	9.77	4.41	0.13	1.60	0.46*	3.28 ns	0.33	9.99	3.20	0.21	1.81	0.89**	4.57*
NPPP	39.26	422.31	192.62	5.31	65.23	0.58**	3.78 ns	42.75	492.50	179.40	9.61	83.80	1.05**	4.82*
SW	1.47	3.50	2.47	0.02	0.25	−0.82**	7.41**	0.47	3.40	2.16	0.06	0.50	0.48 ns	3.93 ns

**SKUAST**														

**Cross ILL10829 × ILWL30**								**Cross ILL8006 × ILWL62**						
	
Trait	Min	Max	Mean	SE	SD	Skewness	Kurtosis	Min	Max	Mean	SE	SD	Skewness	Kurtosis

DF	77.00	125.00	87.52	0.49	6.10	1.77**	11.04**	86.8	140.05	101.62	0.92	8.80	1.58**	9.09**
DM	125.00	170.00	135.08	0.40	4.90	2.46**	18.53**	125.00	170.00	141.19	0.60	5.75	0.98**	8.98**
SYPP	1.22	14.06	5.40	0.27	3.34	1.17**	3.98*	0.01	16.68	4.31	0.42	3.99	1.55**	5.54**
NPPP	48.34	420.84	155.88	6.21	76.86	1.06**	3.94*	3.70	596.20	119.41	10.36	98.84	1.80**	7.79**
SW	1.48	4.71	3.66	0.06	0.77	−0.88**	2.77 ns	2.68	4.74	3.57	0.05	0.44	0.50*	2.76 ns

*Significant at p = 0.01, **Significant at p = 0.05, ns, non-significant.

**FIGURE 2:**
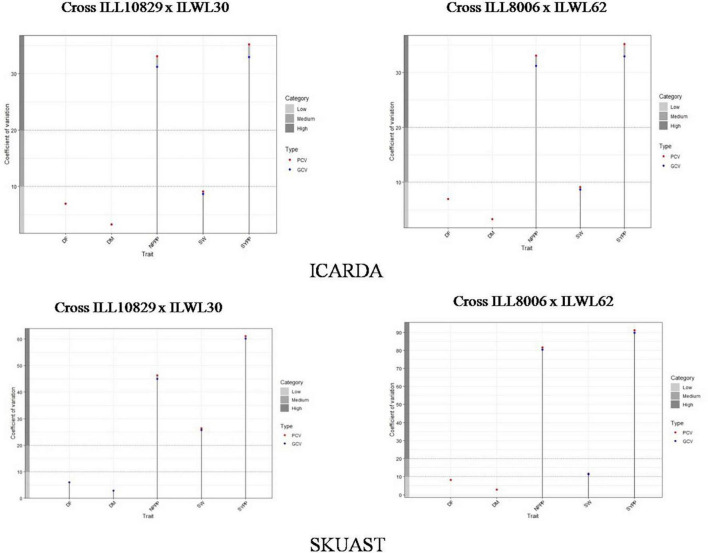
Estimation of the coefficient of variation for lentil interspecific derivatives at ICARDA and SKUAST, India. DF, days to 50% flowering; DM, days to 80% maturity; NPPP, number of pods plant^– 1^; SW, 100-seed weight; SYPP, seed yield plant^– 1^.

Further, it has been seen that the magnitude of the phenotypic coefficient of variation is greater than the genotypic coefficient of variation. Our results revealed that at ICARDA, the coefficient of variation in the cross of ILL10829 × ILWL30 was low for days to 50% flowering, days to 80% maturity and 100-seed weight, and high for the number of pods plant^–1^ and seed yield plant^–1^ (Figure 2). The same trend of variation was also noticed in the cross of ILL8006 × ILWL62 at this location. Likewise, in SKUAST, the coefficient of variation was low for days to 50% flowering and days to 80% maturity, along with high for number of pods plant^–1^ and seed yield plant^–1^ in both the crosses (Figure 2).

### Estimation of broad-sense heritability and adjusted mean boxplot performance

**FIGURE 3:**
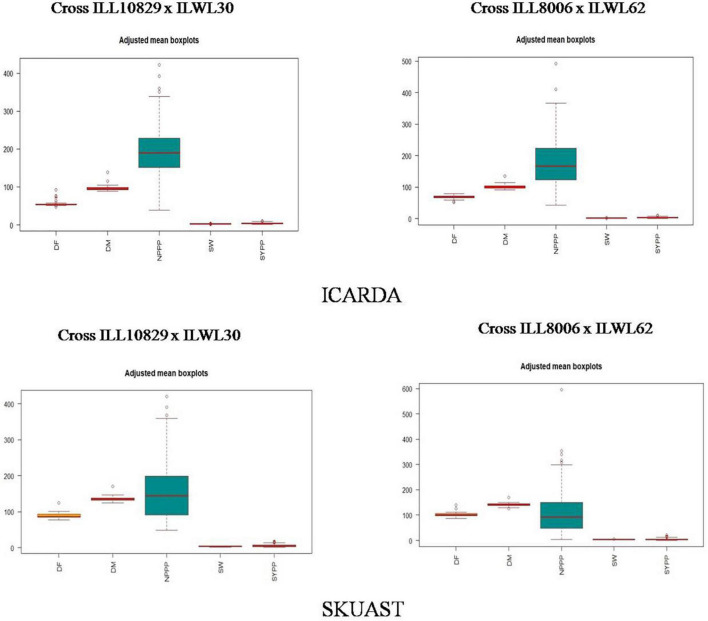
Estimation of adjusted mean boxplots for different traits in lentil interspecific derivatives at ICARDA and SKUAST, India. DF, days to 50% flowering; DM, days to 80% maturity; NPPP, number of pods plant^– 1^; SW, 100-seed weight; SYPP, seed yield plant^– 1^.

The heritability (bs) estimates were high for the number of pods plant^–1^, 100-seed weight, and seed yield plant^–1^ in both the crosses (ILL10829 × ILWL30 and ILL8006 × ILWL62) at ICARDA (Supplementary Figure 1). Likewise, in SKUAST, high heritability in both the crosses was recorded for all the characters except days to 50% flowering and days to 80% maturity in the cross of ILL8006 × ILWL62, where it was very low (Supplementary Figure 1). Further, the results of adjusted means in the form of boxplots for both the crosses manifested almost similar trends for days to 50% flowering, days to 80% maturity, 100-seed weight, and seed yield plant^–1^ at ICARDA (Figure 3), barring the number of pods plant^–1^, which showed a high degree of variability in both crosses. Similar observations were also assessed in SKUAST for the aforementioned characters except for the number of pods plant^–1^, where less variability was recorded in the cross ILL8006 × ILWL62 as compared to the cross ILL10829 × ILWL30 (Figure 3). Furthermore, to consolidate these findings, genotypic, phenotypic, and environmental coefficients of variation also showed a similar sort of pattern for important agronomical traits in both crosses at ICARDA and SKUAST as depicted in Supplementary Table 1. In the cross ILL10829 × ILWL30, high values for the genotypic and phenotypic coefficients of variation were observed for the number of pods plant^–1^, seed yield plant^–1,^ and 100-seed weight at ICARDA, as well as SKUAST. Notably, similar results were also obtained for these characters in the cross ILL8006 × ILWL62 at both locations.

### Correlations and principal component analysis

**FIGURE 4:**
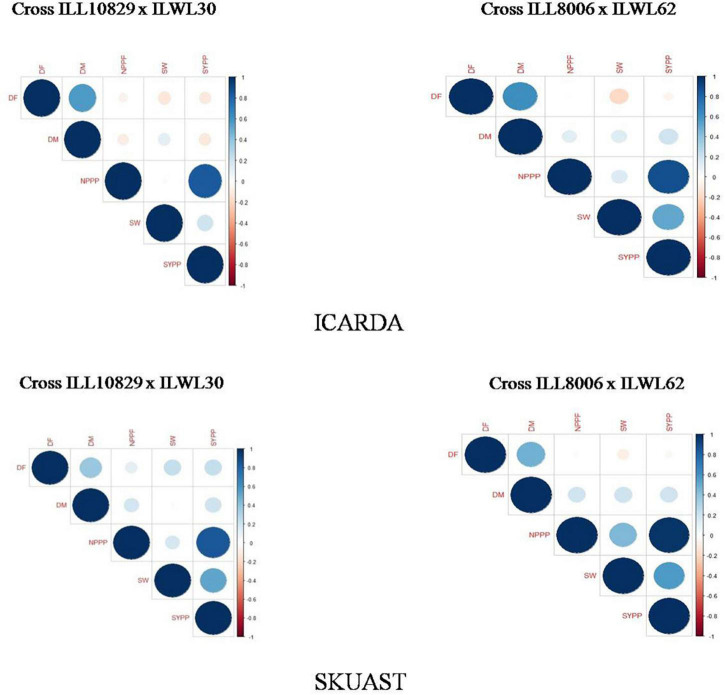
Estimation of correlation plots for agro-morphological traits among lentil interspecific derivatives at ICARDA and SKUAST, India. DF, days to 50% flowering; DM, days to 80% maturity; NPPP, number of pods plant^– 1^; SW, 100-seed weight; SYPP, seed yield plant^– 1^.

The association between the number of pods plant^–1^ and seed yield plant^–1^ showed positive correlations among each other for both the crosses at ICARDA and SKUAST (Figure 4). However, the results obtained using PCA showed a paradoxical pattern for these traits at both locations (Supplementary Figure 2). It was observed that days to 50% flowering and days to 80% maturity showed a negative correlation with each other, while traits like the number of pods plant^–1^ and seed yield plant^–1^ were found to be positively correlated in the cross ILL10829 × ILWL30 at ICARDA. All these traits showed a negative correlation in the cross ILL8006 × ILWL62 at ICARDA. At SKUAST, a negative correlation was observed for all traits in the cross ILL10829 × ILWL30, while a positive correlation existed among days to 50% flowering and days to 80% maturity in the cross ILL8006 × ILWL62.

### Station trials

#### Agronomical evaluation

The best performing interspecific derivatives of SKUAST were selected from the populations and assessed subsequently for three consecutive seasons during the winters of 2016–2017, 2017–2018, and 2018–2019. The results demonstrated that the seed yield of different lentil genotypes differed significantly, and the yield values exhibited a slightly higher trend in winter 2016–2017 compared to the years 2017–2018 and 2018–2019 (Table 4). The genotype Jammu Lentil 144 demonstrated a significantly higher seed yield pooled value (17.00 q/ha) than the check varieties. The tests L4147 and L699 registered significantly lower seed yield pooled values of 8.60 and 10.87 q/ha, respectively. However, check PL406 was statistically at par with Jammu Lentil 144 (Table 4).

**TABLE 4:** Performance of selected derivative Jammu Lentil 144 along with checks during winter seasons of 2016–2017, 2017–2018, and 2018–2019.

Genotype	2016–2017			2017–2018			2018–2019			Pooled		
				
	DF	DM	SY (q/ha)	DF	DM	SY (q/ha)	DF (50%)	DM	SY (q/ha)	DF	DM	SY (q/ha)
Jammu Lentil 144	84.24	131.64	17.67	86.33	131.31	16.83	87.50	130.25	16.50	86.02	131.06	17.00
Check (L4147)	75.74	126.66	9.25	81.90	129.50	8.20	82.15	130.50	8.35	79.93	128.88	8.60
Check (L699)	85.00	125.00	13.50	82.34	141.90	10.07	80.26	132.56	9.06	82.53	133.15	10.87
Check (PL406)	124.00	163.66	15.18	123.66	158.00	14.00	122.02	160.23	14.25	123.22	160.63	14.47
CD%	3.63	4.87	2.25	5.87	4.46	2.12	3.65	4.90	2.26	1.86	2.57	1.23

Jammu Lentil 144 took 86.02 and 131.06 days to 50% flowering and days to 80% maturity, respectively, which was on par with regional check variety L699 (flowering 82.53 days and maturity 133.15 days) (Table 4). Overall, Jammu Lentil 144 exhibited earliness in maturity by 29 days, and seed yield increased by 16.65% as compared to the highest yielding check variety PL406 during winter 2016–2017, 2017–2018, and 2018–2019. Similarly, genotype Jammu Lentil 71 exhibited a 9.40% higher seed yield (23.74 q/ha) over check L699 (21.70 q/ha). It also showed earliness by 15 days as compared to check PL406 under rainfed conditions (Table 5). As a result, both these genotypes were recommended for commercial cultivation in the rainfed area of the Jammu region.

**TABLE 5:** Performance of selected derivative Jammu Lentil 71 along with checks during winter seasons of 2016–2017, 2017–2018, and 2018–2019.

Genotype	2016–2017			2017–2018			2018–2019			Pooled		
				
	DF	DM	SY (q/ha)	DF	DM	SY (q/ha)	DF	DM	SY (q/ha)	DF	DM	SY (q/ha)
Jammu Lentil 71	102.80	142.33	25.36	107.66	149.07	25.61	105.25	145.00	20.25	105.24	145.46	23.74
Check (L699)	100.00	142.33	24.36	102.33	144.00	22.00	101.40	140.00	18.75	101.24	142.11	21.70
Check (PL406)	123.33	162.75	17.53	122.00	159.31	15.30	121.23	160.00	16.50	122.18	160.68	16.44
CD 5%	4.40	6.83	3.83	3.98	6.42	2.19	4.42	6.85	3.85	3.28	4.73	2.06

#### Screening against major biotic stresses

Jammu Lentil 144 was also screened against major biotic stresses, *viz.* fusarium wilt, root rot, pod borer, and aphids during the winter seasons of 2016–2017, 2017–2018, and 2018–2019. The pooled analysis revealed that Jammu Lentil 144 showed a resistant reaction to fusarium wilt and root rot infestations. However, it showed low susceptibility and moderately resistant reactions to pod borer and aphids, respectively (Table 6). Similarly, check L699 also demonstrated parallel trends against the aforementioned biotic stresses, while L4147 showed resistance against all of them. Further, the performance of Jammu Lentil 71 was found to be superior over checks for all biotic stresses as it showed resistance to all of them. Although check L699 was found to be resistant to fusarium wilt and root rot, PL406 exhibited low to moderate susceptibility to all of them, including pod borer and aphids (Table 7).

**TABLE 6:** Screening of selected derivative Jammu Lentil 144 against major biotic stresses during winter seasons of 2016–2017, 2017–2018, and 2018–2019.

Genotype	Pooled data on fusarium wilt incidence (%)		Pooled data on root rot (%)		Pooled data on lentil pod borer (% pod damage)		Pooled data on aphids (no. of aphids/plant)	
				
	Mean	Reaction	Mean	Reaction	Mean	Reaction	Mean	Reaction
Jammu Lentil 144	2.45	R	3.50	R	3.05	LS	2.16	MR
Check (L4147)	3.51	R	4.25	R	0.85	R	1.64	R
Check (L699)	4.66	R	5.25	R	1.70	LS	5.05	MR
Check (PL406)	17.38	MS	15.75	MS	16.10	MS	23.26	MS

**TABLE 7:** Screening of selected derivative Jammu Lentil 71 against major biotic stresses during winter seasons of 2016–2017, 2017–2018, and 2018–2019.

Genotype	Pooled data on fusarium wilt incidence (%)		Pooled data on root rot (%)		Pooled data on lentil pod borer (% pod damage)		Pooled data on aphids (no. of aphids/plant)	
				
	Mean	Reaction	Mean	Reaction	Mean	Reaction	Mean	Reaction
Jammu Lentil 71	3.05	R	4.25	R	0.93	R	1.66	R
Check (L699)	2.55	R	5.25	R	3.84	LS	4.22	MR
Check (PL406)	17.38	MS	15.75	MS	8.74	LS	23.26	MS

#### Estimation of promising cultivars with different nutrient levels

The performance of the selected derivative, Jammu Lentil, 144 of cross ILL10829 × ILWL30, was assessed under different doses of fertilizers having desired concentrations of macronutrients like nitrogen (N) and phosphorus (P). A significantly higher seed yield (14.69 q/ha) was observed with a 25% higher fertilizer dose, followed by the seed yields obtained by the treatments having the recommended dose (13.37 q/ha). However, the treatments receiving a 25% higher fertilizer dose also took more days to 50% flowering (99.7) and more days to 80% maturity (141.4) than other doses of fertilizer (Table 8). Moreover, a similar trend was also observed for Jammu Lentil 71 of cross ILL8006 × ILWL62, where 18.81 q/ha seed yield was recorded at a 25% higher fertilizer dose along with more days to 50% flowering (110) and more days to 80% maturity (147.1) (Table 9).

**TABLE 8:** Performance of selected derivative Jammu Lentil 144 at different nutrient levels under rainfed conditions of Jammu region.

Treatments/varieties	Winter 2017–2018			Winter 2018–2019			Pooled		
			
	DF	DM	SY (q/ha)	DF	DM	SY (q/ha)	DF	DM	SY (q/ha)
**Entries/varieties**									
V_1_: Jammu Lentil 144	85.9	132.6	16.65	84.7	131.3	15.74	85.3	132.0	16.19
V_2_: Check (L4147)	82.3	130.0	8.79	80.4	128.7	8.30	81.3	129.4	8.55
V_3_: Check (L699)	99.0	139.2	14.37	97.6	138.0	13.37	98.3	138.6	13.87
V_4_: Check (PL406)	124.7	158.1	14.15	123.4	156.6	13.35	124.1	157.4	13.75
CD (5%)	–	–	1.09	–	–	0.93	–	–	1.01
**Nutrient levels**									
N_1_: 25% less than recommended NP	95.1	137.8	11.60	93.7	136.1	10.83	94.4	136.9	11.21
N_2_: Recommended NP	98.4	140.3	13.71	97.0	139.0	13.02	97.7	139.6	13.37
N_3_: 25% more than recommended NP	100.5	141.9	15.17	98.8	140.9	14.21	99.7	141.4	14.69
CD (5%)	–	–	0.95	–	–	0.80	–	–	0.82
**Entries × Nutrient levels**									
CD (5%)	–	–	NS	–	–	NS	–	–	NS

**TABLE 9:** Performance of selected derivative Jammu Lentil 71 at different levels of nutrients under rainfed conditions of Jammu region.

Treatments/varieties	Winter 2017–2018			Winter 2018–2019			Pooled		
			
	DF	DM	SY (q/ha)	DF	DM	SY (q/ha)	DF	DM	SY (q/ha)
**Entries/varieties**									
V_1_: Jammu Lentil 71	108.8	149.0	21.71	108.7	148.5	20.67	108.8	148.7	21.19
V_2_: Check (L699)	102.9	144.6	17.58	101.6	145.3	16.82	102.3	145.0	17.20
V_3_: Check (PL406)	123.2	159.8	15.42	124.7	159.0	14.60	124.0	159.4	15.01
CD (5%)	–	–	1.46	–	–	1.72	–	–	1.59
**Nutrient levels**									
N_1_: 25% less than recommended NP	102.0	144.8	15.82	102.1	144.2	13.03	102.1	144.5	14.42
N_2_: Recommended NP	106.0	145.9	19.09	106.6	146.0	16.16	106.3	146.0	17.62
N_3_: 25% more than recommended NP	110.2	147.2	21.19	109.9	147.0	18.44	110.0	147.1	19.81
CD (5%)	–	–	1.26	–	–	1.49	–	–	1.37
**Entries × Nutrient levels**									
CD (5%)	–	–	NS	–	–	NS	–	–	NS

Likewise, these promising derivatives were also assessed according to standard agronomical practices of the Jammu region during the winters of 2017–2018 and 2018–2019. Our results revealed a significantly higher seed yield of both Jammu Lentil 144 (13.28 q/ha) and Jammu Lentil 71 (18.08 q/ha) during the 20th October sowing, followed by the 4th and 19th November sowings, respectively. Interestingly, both took more days to 50% flowering and more days to 80% maturity during early sowing (20th October) (Supplementary Tables 2, 3).

## Discussion

Crop wild relatives (CWRs) are vital resources for providing a pool of genetic diversity that can be utilized for enhancing genetic gains and breeding new and adapted crop varieties resistant to prevailing stresses and other factors (Brozynska et al., 2016). The utilization of CWRs for broadening the genetic base of domesticated germplasm is attaining significant progress in the identification of novel genes and alleles to improve the yield levels of existing crop varieties (Hunter and Heywood, 2011; Bohra et al., 2021). In this study, we have assessed the agronomic performance of advanced lentil interspecific derivatives obtained from *L. culinaris* ssp. *orientalis*, and *L. ervoides* under two agroecological regions of India. The study revealed that analysis of variance exhibited significant variations for important agro-morphological traits like days to 80% maturity, the number of pods plant^–1,^ and seed yield plant^–1^ among lentil interspecific derivatives at ICARDA and SKUAST, India. This could have occurred due to the expression of atypical recessive alleles (Rick and Smith, 1953) or complementary gene action (Vega and Frey, 1980) in advanced generations of interspecific crosses of ILL10829 × ILWL30 and ILL8006 × ILWL62 evaluated at the above locations. Moreover, the estimation of genotypic and phenotypic coefficients of variation and adjusted mean boxplots demonstrated identical variations for the number of pods plant^–1^, seed yield plant^–1^, and 100-seed weight. High heritability in conjunction with high genetic advance was noticed for the majority of characters in both crosses, indicating fixation of additive gene effects, resulting in better selection of genetic materials with desired traits (Singh et al., 2017). Succeeding reports by Xu et al. (2017) and Hamidou et al. (2018) on the importance of genetic variability for yield and its contributing traits have also demonstrated this scenario very well. Nonetheless, days to 80% maturity revealed low heritability values, suggesting that the character is greatly influenced by environmental effects and selection would not be much useful. However, the pattern analysis indicated that the most important characteristics with higher weight to total variation are the number of pods plant^–1^ and seed yield plant^–1,^ suggesting their role in broadening the genetic base of the cultivated gene pool. Our findings are supported by the results of Haddad and Muehlbaurer (1991), where three lentil interspecific populations were advanced from F_2_ to F_4_ generations by single seed descent (SSD) and bulk population (BP) breeding methods that compared the relative efficiency for maintaining genetic variability and desirable selection. They also indicated that the SSD method maintained more genetic variability than BP for most of the traits assessed. Furthermore, the best performing interspecific derivatives selected from total populations of both crosses at SKUAST, India, named “Jammu Lentil 144” and “Jammu Lentil 71” were assessed consecutively for three seasons, and performance was also compared with local and national checks. The data indicated that Jammu Lentil 144 (16.65%) and Jammu Lentil 71 (9.40%) generated a high yield as compared to check varieties, including earliness by 25 and 15 days, respectively. Keeping this substantial genetic improvement in view, these promising derivatives resulted in the identification of potential entries for developing suitable cultivars in the rainfed area of the Jammu region in India. The significantly higher seed yield of both entries over the others could be due to their adaptability and better suitability in the target region. Singh et al. (2011) and Mian et al. (2016) also observed significant seed yield increase values among different lentil varieties. Further, the screening of both selected entries against diseases and insects indicated that disease incidence was sufficient for the identification and selection of resistant types. Both Jammu Lentil 144 and Jammu Lentil 71 showed resistance against fusarium wilt and root rot, although the latter was also found to be resistant to pod borer and aphids. This has confirmed that genetic resistance has been transferred from wild *Lens* species to a cultivated gene pool using the pre-breeding approach. These findings suggest that they would be useful cultivars for their commercialization by farming communities in the target area. As far as standard agronomic practices of these selected entries are concerned, both showed a remarkable pattern of expression at a 25% higher dose of nutrient levels. Researchers like Singh et al. (2003), Khan et al. (2006), and Rasheed et al. (2010) have also reported that N and P are essential for obtaining desirable yield levels. Mahmood et al. (2011) also observed significantly higher lentil seed yields with increasing doses of N and P under rainfed conditions. Our results also demonstrated significant seed yield of both these entries during the early sowing period (20th October) in rainfed regions of the Jammu region. This could have been because the early sown crop escapes the higher temperatures that might have prevailed during the grain filling and reproductive stages of the lentil crop. Similar types of findings were also reported by Mian and Islam (2010) and Ouji and Mouelhi (2017).

For their consistency in the aforementioned period and selected trials, both Jammu Lentil 144 and Jammu Lentil 71 were recommended for commercial cultivation in the rainfed regions of Jammu. Lastly, it is summarized that significant agronomic improvement and disease resistance were observed from advanced pre-breeding materials derived from *L. culinaris* ssp. *orientalis* and *L*. *ervoides* wild lentil taxa. It is noteworthy that some potential interspecific derivative lines performed better than local and national checks, which were, hence, recommended for their commercial farming in the Jammu region of India. The other useful genetic materials are also being advanced for further fruitful selection at ICARDA. These cultivars could be utilized as a potential resource of various untapped genes controlling enviable traits for biotic and abiotic stresses along with high yield to substantially improve the productivity of cultivated lentil growing elsewhere.

## Data availability statement

The original contributions presented in this study are included in the article/Supplementary material, further inquiries can be directed to the corresponding author.

## Author contributions

MS: conceptualization, supervision, and writing—original draft. SK and RM: investigation, methodology, and formal analysis. SS and NM: data validation and writing—review and editing. RS, SJ, and VG: formal analysis and data validation. All authors contributed to the article and approved the submitted version.

## Abbreviations

ICARDA: International Centre for Agricultural Research in Dry Areas
SKUAST: Sher-e-Kashmir University of Agricultural Sciences and Technology
ACBD: augmented complete block design
ILL: International Legume Lentil
ILWL: International Legume Wild Lentil
ANOVA: analysis of variance
PCA: principal component analysis
DF: days to 50% flowering
DM: days to 80% maturity
NPPP: number of pods plant^–1^
SYPP: seed yield plant^–1^
SW: seed weight
SSD: single seed descent
PCA: principal component analysis
REML: restricted maximum likelihood.
