# Supplementary material for: Evaluation and identification of advanced lentil interspecific derivatives resulted in the development of early maturing, high yielding, and disease-resistant cultivars under Indian agro-ecological conditions
Source: Front Plant Sci. 2022 Sep 8;13:936572. doi: 10.3389/fpls.2022.936572 (PMC9499259; doi:10.3389/fpls.2022.936572)
Supplement: Supplementary file 1 [file Data_Sheet_1.docx]

Cross ILL10829 x ILWL30 Cross ILL8006 x ILWL62


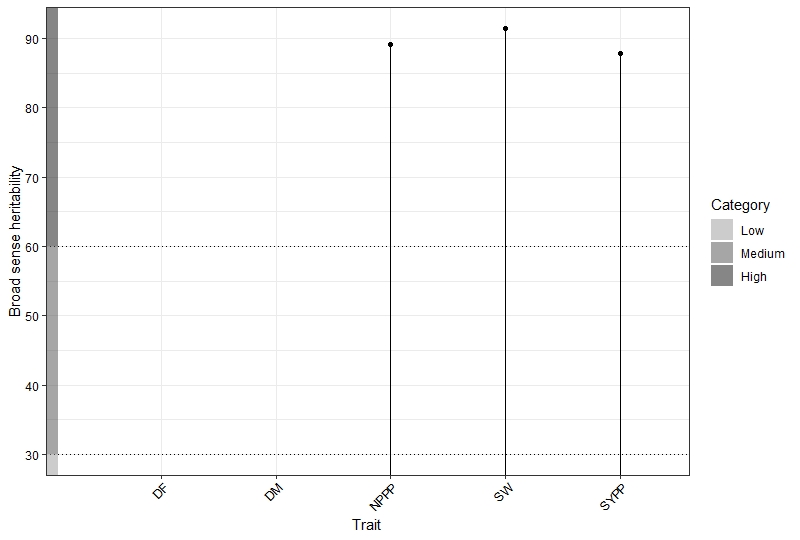

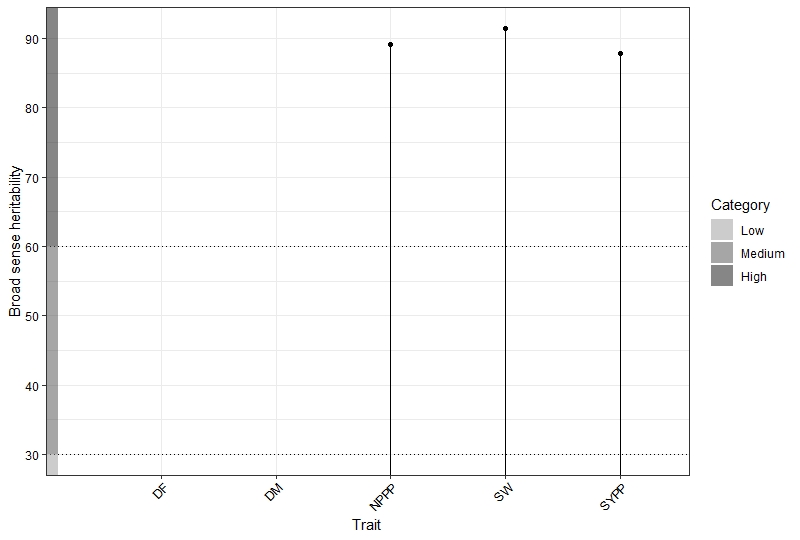


ICARDA

Cross ILL10829 x ILWL30   Cross ILL8006 x ILWL62


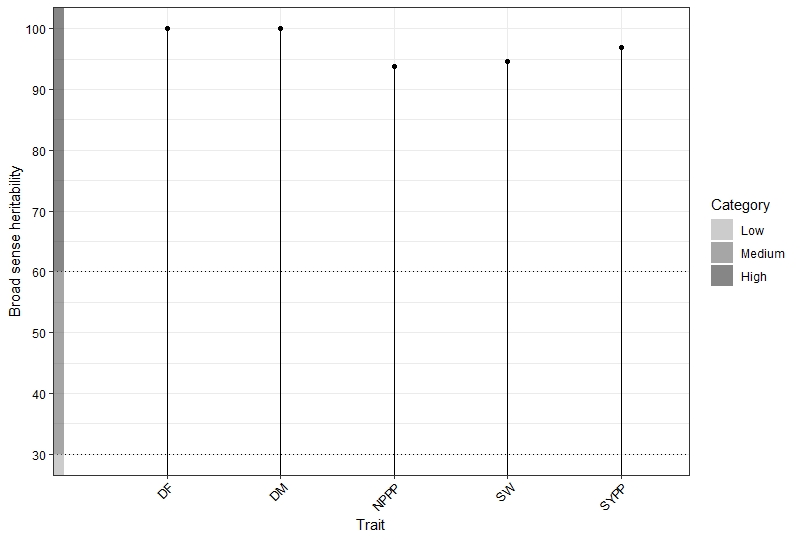

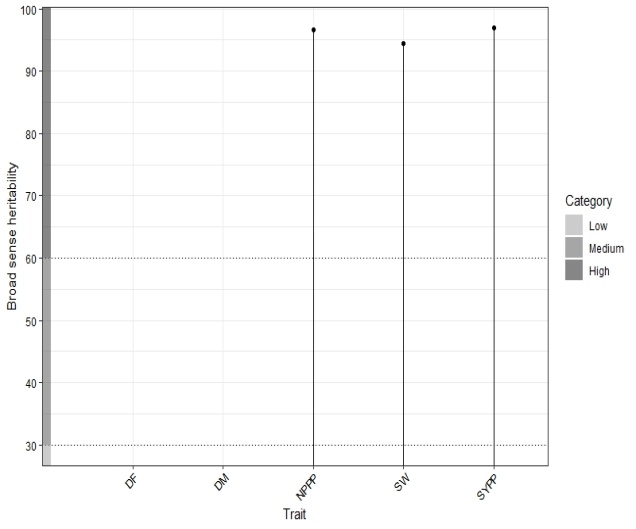


SKUAST

**Supplementary Fig. 1.** Broad sense heritability for lentil interspecific derivatives at ICARDA and SKUAST, India.

Cross ILL10829 x ILWL30 Cross ILL8006 x ILWL62


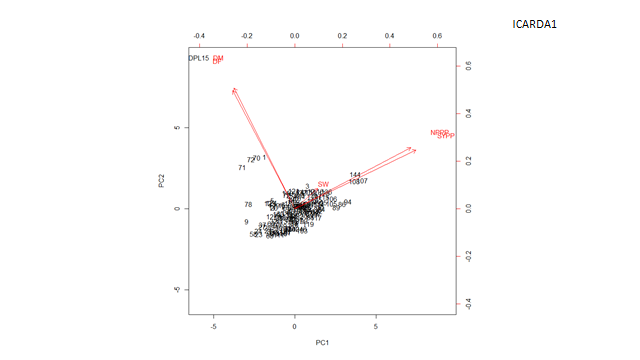

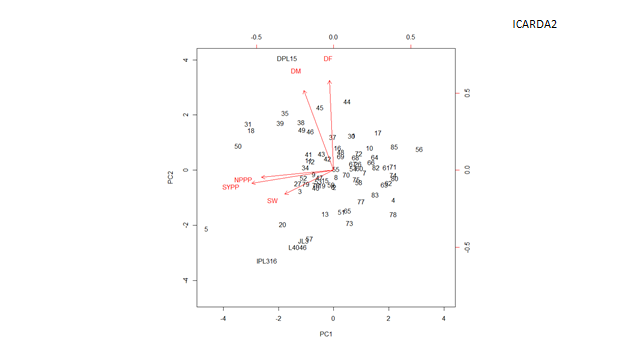


Cross ILL10829 x ILWL30   Cross ILL8006 x ILWL62


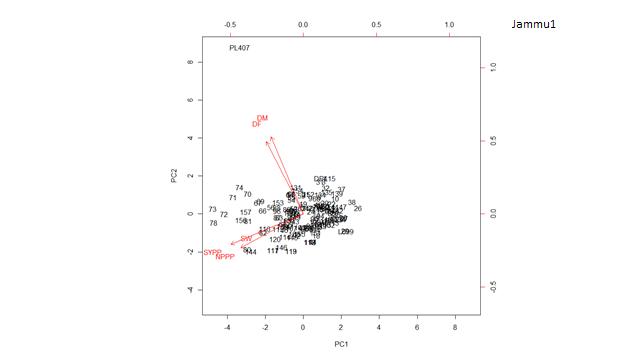

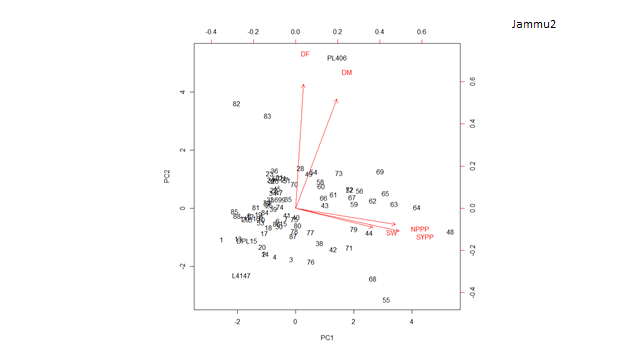


**Supplementary Fig. 2.** PCA biplots for lentil interspecific derivatives at ICARDA and SKUAST, India.

**Supplementary Table 1** Mean, variance, phenotypic, genotypic and environmental coefficient of variance, heritability, genetic advance and genetic advance as percent of mean for important traits.

| **ICARDA** | | | | | | | | | | |
| --- | --- | --- | --- | --- | --- | --- | --- | --- | --- | --- |
| Cross ILL10829 x ILWL30 | | | | | | | | | | |
| Trait | Mean | GV | PV | EV | GCV | PCV | ECV | H (bs) | GA | GAM |
| DF | 54.96 | - | 14.59 | 24.92 | NA | 6.95 | 9.08 | - | - | - |
| DM | 96.18 | - | 9.99 | 15.28 | NA | 3.29 | 4.06 | - | - | - |
| SYPP | 4.41 | 2.11 | 2.40 | 0.29 | 32.98 | 35.19 | 12.26 | 87.86 | 2.81 | 63.78 |
| NPPP | 192.62 | 3616.41 | 4059.54 | 443.12 | 31.22 | 33.08 | 10.93 | 89.08 | 117.10 | 60.79 |
| SW | 2.47 | 0.05 | 0.05 | 0.00 | 8.70 | 9.09 | 2.66 | 91.42 | 0.42 | 17.15 |
| Cross ILL8006 x ILWL62 | | | | | | | | | | |
| Trait | Mean | GV | PV | EV | GCV | PCV | ECV | H (bs) | GA | GAM |
| DF | 68.86 | - | 19.18 | 26.86 | NA | 6.36 | 7.53 | - | - | - |
| DM | 100.61 | - | 12.30 | 25.56 | NA | 3.49 | 5.03 | - | - | - |
| SYPP | 3.20 | 1.53 | 2.55 | 1.02 | 38.63 | 49.86 | 31.53 | 60.02 | 1.98 | 61.74 |
| NPPP | 179.40 | 3355.79 | 6768.51 | 3412.72 | 32.29 | 45.86 | 32.56 | 49.58 | 84.15 | 46.90 |
| SW | 1.93 | 0.06 | 0.12 | 0.07 | 12.29 | 18.30 | 13.57 | 45.07 | 0.33 | 17.02 |
| **SKUAST** | | | | | | | | | | |
| Cross ILL10829 x ILWL30 | | | | | | | | | | |
| Trait | Mean | GV | PV | EV | GCV | PCV | ECV | H (bs) | GA | GAM |
| DF | 87.52 | 28.46 | 28.46 | 2.30E-28 | 6.10 | 6.10 | 1.70E-14 | 100.00 | 11.01 | 12.57 |
| DM | 135.08 | 15.44 | 15.44 | 1.10E-28 | 2.91 | 2.91 | 7.90E-15 | 100.00 | 8.11 | 6.00 |
| SYPP | 5.40 | 10.55 | 10.88 | 0.33 | 60.16 | 61.10 | 10.71 | 96.93 | 6.60 | 122.18 |
| NPPP | 155.88 | 4909.14 | 5231.19 | 322.06 | 44.95 | 46.40 | 11.51 | 93.84 | 140.02 | 89.83 |
| SW | 3.66 | 0.88 | 0.93 | 0.05 | 25.70 | 26.43 | 6.16 | 94.56 | 1.89 | 51.56 |
| Cross ILL8006 x ILWL62 | | | | | | | | | | |
| Trait | Mean | GV | PV | EV | GCV | PCV | ECV | H (bs) | GA | GAM |
| DF | 87.52 | 28.46 | 28.46 | 2.30E-28 | 6.10 | 6.10 | 1.70E-14 | 100.00 | 11.01 | 12.57 |
| DM | 135.08 | 15.44 | 15.44 | 1.10E-28 | 2.91 | 2.91 | 7.90E-15 | 100.00 | 8.11 | 6.00 |
| SYPP | 5.40 | 10.55 | 10.88 | 0.33 | 60.16 | 61.10 | 10.71 | 96.93 | 6.60 | 122.18 |
| NPPP | 155.88 | 4909.14 | 5231.19 | 322.06 | 44.95 | 46.40 | 11.51 | 93.84 | 140.02 | 89.83 |
| SW | 3.66 | 0.88 | 0.93 | 0.05 | 25.70 | 26.43 | 6.16 | 94.56 | 1.89 | 51.56 |

PV, phenotypic variance; GV, genotypic variance; EV, extreme variability; GCV, genotypic coefficient of variation; PCV, phenotypic coefficient variation; ECV, extreme climate variability; H(bs), heritability in broad sense; GA, genetic advance; GAM, genetic advance over mean

**Supplementary Table 2** Performance of selected derivative Jammu Lentil 144 at different sowing dates under rainfed conditions of Jammu region.

| Genotype | Winter2017-18 | | | | Winter2018-19 | | | Pooled | | |
| --- | --- | --- | --- | --- | --- | --- | --- | --- | --- | --- |
|  | DF | DM | GY (q/ha) | | DF | DM | GY (q/ha) | DF | DM | GY (q/ha) |
| Entries | | | | | | | | | | |
| V_1_: Jammu Lentil144 | 84.0 | 129.0 | | 14.42 | 82.5 | 127.3 | 13.67 | 83.3 | 128.2 | 14.05 |
| V_2_: Check (L4147) | 78.9 | 123.7 | | 6.87 | 76.9 | 122.5 | 6.22 | 77.9 | 123.1 | 6.54 |
| V_3_: Check (L699) | 94.0 | 133.5 | | 12.49 | 94.7 | 132.6 | 12.01 | 94.3 | 133.1 | 12.25 |
| V_4_: Check (PL406) | 118.6 | 153.7 | | 12.26 | 119.3 | 153.7 | 11.78 | 118.9 | 153.7 | 12.02 |
| CD (5%) |  |  | | 1.06 |  |  | 1.08 |  |  | 1.07 |
| Sowing dates | | | | | | | | | | |
| D_1_: 20^th^ October | 98.3 | 139.5 | | 13.66 | 98.1 | 138.4 | 12.91 | 98.2 | 139.0 | 13.28 |
| D_2_: 04^th^ November | 93.6 | 135.1 | | 11.47 | 93.6 | 134.4 | 10.95 | 93.6 | 134.7 | 11.21 |
| D_3_: 19^th^ November | 89.7 | 130.3 | | 9.40 | 88.4 | 129.3 | 8.86 | 89.0 | 129.8 | 9.13 |
| CD (5%) |  |  | | 0.92 |  |  | 0.93 |  |  | 0.92 |
| Entries x Sowing dates | | | | | | | | | | |
| CD (5%) | **-** | **-** | | NS | - | - | NS | - | - | NS |

**Supplementary Table 3** Performance of selected derivative Jammu Lentil 71 at different sowing dates under rainfed conditions of Jammu region.

| Genotype | Winter2017-18 | | | Winter 2018-19 | | | Pooled | | |
| --- | --- | --- | --- | --- | --- | --- | --- | --- | --- |
|  | DF | DM | GY (q/ha) | DF | DM | GY (q/ha) | DF | DM | GY (q/ha) |
| Entries | | | | | | | | | |
| V_1_: Jammu Lentil 71 | 101.3 | 141.0 | 19.96 | 100.9 | 139.4 | 19.08 | 101.1 | 140.2 | 19.52 |
| V_2_: Check (L699) | 95.6 | 136.4 | 16.13 | 95.4 | 135.3 | 15.17 | 95.5 | 135.8 | 15.65 |
| Check (PL406) | 115.5 | 151.2 | 13.46 | 114.0 | 149.2 | 13.15 | 114.7 | 150.2 | 13.30 |
| CD (5%) |  |  | 1.13 |  |  | 1.26 |  |  | 1.19 |
| Sowing dates | | | | | | | | | |
| D_1_: 20^th^ October | 106.6 | 146.1 | 18.43 | 105.6 | 144.4 | 17.72 | 106.1 | 145.2 | 18.08 |
| D_2_: 04^th^ November | 99.6 | 138.8 | 15.56 | 99.9 | 138.3 | 14.91 | 99.7 | 138.5 | 15.23 |
| D_3_: 19^th^ November | 90.6 | 129.7 | 10.68 | 90.2 | 129.2 | 9.76 | 90.3 | 129.4 | 10.22 |
| CD (5%) |  |  | 0.98 |  |  | 1.09 |  |  | 1.03 |
| Entries x Sowing dates | | | | | | | | | |
| CD (5%) |  |  | 1.96 |  |  | NS |  |  | 1.76 |
